# Supplementary material for: Patterns of Intron Gain and Loss in Fungi
Source: PLoS Biol. 2004 Nov 30;2(12):e422. doi: 10.1371/journal.pbio.0020422 (PMC532390; doi:10.1371/journal.pbio.0020422)
Supplement: Table S1 — Also available at http://genes.mit.edu/NielsenEtAl/. (4.3 MB ZIP). [file pbio.0020422.st001.zip › NielsenEtAl/html/1299.html]

AN0843.1.NCU09475.1.MG06658.1.FG06019.1


```
 CLUSTAL W (1.82) Multiple Sequence Alignments - Introns Inserted


Sequence 1: NCU09475.1	193 aa
Sequence 2: MG06658.1	187 aa
Sequence 3: FG06019.1	181 aa
Sequence 4: AN0843.1	189 aa
Alignment Length: 194 aa
Number Identitical Residues: 148 aa
Alignment Score (without introns) 5606


MG06658.1 	-------MRQETHDASNPKADI~GHLLSR~DYIQIRSPVYIPHSAGRYAVKRFRKANCPI
NCU09475.1	MGNIHLGRKDDTRHIRERRAND~QYLRS-~DYIQIRSPVYLPHSAGRYAAKRFRKANCPI
FG06019.1 	-------MSDVAVDGSVKTTGH2------~DYISLRNPVYVTHTAGRYATKRFRKANCPI
AN0843.1  	-----MSKKHFSRPIANQRNRP~THLTNP2DYIQIRSPVYLPHTAGRYAAKRFRKAQCPI
          	      .  . :               .  ***.:*.***:.*:*****.******:***

MG06658.1 	IERLTNSLMMHGRNNGKKLMAVRIVAHAFEI0IHLMTDQNPIQIAVDAIVNCGPREDSTR
NCU09475.1	IERLTNSLMMHGRNNGKKLMAVRIVAHAFEI0IHLMTDQNPIQIAVDAIVNCGPREDSTR
FG06019.1 	IERLTNSLMHHGRNNGKKLMAVRIVAHAFEI0IHLMTDQNPIQVAVDAIVNCGPREDSTR
AN0843.1  	IERLTNSIMMNGRNNGKKLMAVRIVAHAFEI0IHIMTDQNPLQVAVDAIVNCGPREDSTR
          	*******:* :******************** **:******:*:****************

MG06658.1 	IGSAGTVRRQAVDVSPLRRVNQAISLLTTGAREASFRNVKSIAECLAEELINAAKGSSNS
NCU09475.1	IGSAGTVRRQAVDVSPLRRVNQAIALLTTGAREASFRNVKSIAECLAEELINAAKGSSNS
FG06019.1 	IGSAGTVRRQAVDVSPLRRVNQAISLLTTGAREASFRNVKSIAECLAEELINAAKGSSNS
AN0843.1  	IGSAGTVRRQAVDVSPLRRVNQSIALLTIGAREASFRNIKSIAECLAEELINAAKGSSNS
          	**********************:*:*** *********:*********************

MG06658.1 	YAIKKKDELERVAKSNR
NCU09475.1	YAIKKKDELERVAKSNR
FG06019.1 	YAIKKKDELERVAKSNR
AN0843.1  	YAIKKKDELERVAKSNR
          	*****************
```
